# Audit of Orthopaedic Surgical Documentation

**DOI:** 10.1155/2015/782720

**Published:** 2015-08-19

**Authors:** Fionn Coughlan, Prasad Ellanti, Cliodhna Ní Fhoghlu, Andrew Moriarity, Niall Hogan

**Affiliations:** St. James's Hospital, Dublin, Ireland

## Abstract

*Introduction*. The Royal College of Surgeons in England published guidelines in 2008 outlining the information that should be documented at each surgery. St. James's Hospital uses a standard operation sheet for all surgical procedures and these were examined to assess documentation standards.* Objectives*. To retrospectively audit the hand written orthopaedic operative notes according to established guidelines.* Methods*. A total of 63 operation notes over seven months were audited in terms of date and time of surgery, surgeon, procedure, elective or emergency indication, operative diagnosis, incision details, signature, closure details, tourniquet time, postop instructions, complications, prosthesis, and serial numbers.* Results*. A consultant performed 71.4% of procedures; however, 85.7% of the operative notes were written by the registrar. The date and time of surgery, name of surgeon, procedure name, and signature were documented in all cases. The operative diagnosis and postoperative instructions were frequently not documented in the designated location. Incision details were included in 81.7% and prosthesis details in only 30% while the tourniquet time was not documented in any.* Conclusion*. Completion and documentation of operative procedures were excellent in some areas; improvement is needed in documenting tourniquet time, prosthesis and incision details, and the location of operative diagnosis and postoperative instructions.

## 1. Introduction

Accurate and detailed operation notes are of great importance in all surgical specialities not only for safe patient care but also for providing information for research, audit, and medicolegal purposes [1]. The Royal College of Surgeons Good Surgical Practice guidelines published in 2008 set the standard for all practicing surgeons. These have been updated in 2014 [2]. Operative notes are often presented in legal malpractice cases, and studies have shown that up to 45 percent of operative notes are indefensible medicolegally. Incomplete and illegible notes are a potential source of weakness in a surgeon's defence [3].

Clear, concise, and legible notes are therefore crucial following all surgical procedures. This is difficult to achieve with handwritten notes, especially in the context of legibility. Sweed et al. found that 20 percent of their orthopaedic operation notes contained illegible parts [4]. The new 2104 guidelines now suggest that all notes should “preferably” be “typed.”

St. James's Hospital uses a standard operation sheet for all surgical procedures. The orthopaedic operation notes were examined to assess documentation standards. There are 3 studies which audited operation notes for elective total knee replacements in accordance with the British Orthopaedic Association guidelines [5–7]. Our study examined our operation notes based on the recommendations found in the Royal College of Surgeons of England Good Surgical Practice Guide (2008) [2]. Only 1 previous study has used these same guidelines to audit its orthopaedic operation notes [4].

## 2. Objectives

To retrospectively audit 63 operation notes of inpatients under the care of the orthopaedic service in St. James's Hospital from 9 April 2014 to 21 October 2014 according to the Royal College of Surgeons of England Good Surgical Practice guidelines in February 2008.

## 3. Methods

**Figure 1:**
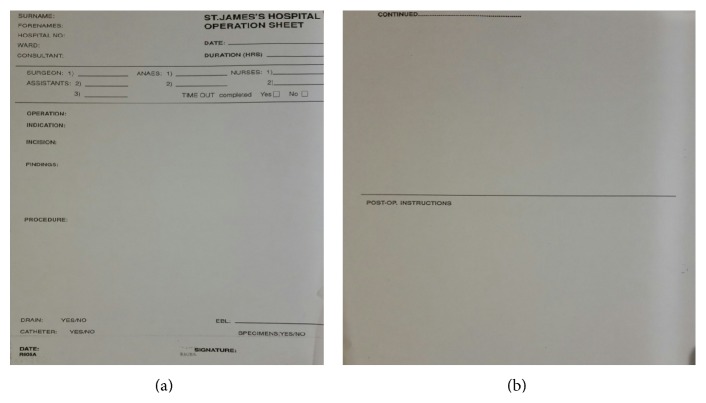
St. James's Hospital operation proforma: front and back.

A total of 63 operation notes were audited by one single reviewer. The operation notes all were based on the standard template (Figure 1) found in St. James's Hospital for all surgical procedures. St. James's operation sheet contains headings for patient details, time and date, duration (hours), surgeon, assistants, anaesthetists, nurses, timeout completed (yes/no), operation, indication, incision, findings, procedure, drain (yes/no), catheter (yes/no), specimen (yes/no), and post-op instructions. The notes were audited in accordance with the College of Surgeons guidelines in terms of date and time of surgery, surgeon, procedure, elective or emergency indication, operative diagnosis, incision details, signature, closure details, tourniquet time, post-op instructions, complications, prostheses, and serial numbers.

## 4. Results

All 63 notes were handwritten on St. James's Hospital standard operation sheet. 71.4% (*n* = 45) of the 63 operations were performed by a consultant surgeon versus 28.6% (*n* = 18) that were performed by trainee registrars. The majority of the operative notes were written by the registrar (85.7%; *n* = 54), followed by the consultant (11.1%; *n* = 7) and the senior house officer (3.17%; *n* = 2). A total of 38.1% (*n* = 24) of all operative notes were written by the lead surgeon, with 61.9% (*n* = 39) written by an assistant. Of the 24 operative notes written by the lead surgeon, 29.1% (*n* = 7) were done by a consultant versus 71% (*n* = 17) by the registrar. All 63 operative notes were handwritten on St. James's Hospital operation sheets. All of the operative notes included date and time of surgery, name of lead surgeon (and any assistants if present), procedure name, and signature. Operative diagnosis was present in 74.6% of the operation notes; however, it was only found in the designated location 63.8% of the time. Incision details were included in 81.7% of the sheets; however, 3 procedures were closed and did not require an incision. Tourniquets were applied in 23.8% of the procedures with none having a documented tourniquet time (0%). Closure details were documented in all but one procedure (98.3%). Postoperative instructions were included in 96.3% of the operative notes, but 41% were located in the incorrect location on the operative sheet. 50 procedures involved the use of prosthetic equipment; however, only 30% of these had documented or attached serial number adhesives to the operation sheet. None of the operative sheets stated whether it was an elective or emergency procedure.

## 5. Discussion

The Royal College of Surgeons of England Good Surgical Practice guidelines help the surgeon create concise, clear, and informative operation notes. This not only allows for better patient care postoperatively but also protects the surgeon medicolegally. Having the proforma operation sheet ensures that the minimum information required is present in all notes and it has been shown to be effective in improving the standard of operation notes [7]. There is only 1 operation sheet template shared among all specialties in St. James's Hospital and therefore it does not allow for the specifics pertaining to different specialities. In orthopaedic surgery, documentation of operation details could be improved with the addition of specific headings for tourniquet application and time, as well as antibiotics used at induction. These were included in the Sheffield proforma and led to better completion of detailed notes [7]. The benefits of proforma have been documented with other specialities such as pediatric surgery [8] and maxillofacial surgery [9].

Singh et al. have shown that audits such as these can significantly improve the quality of the operative notes by simply highlighting the deficient areas [10]. Others have suggested the use of a checklist as an additional tool to improve the quality of the operative notes [5].

A similar study to ours was conducted by Sweed et al. in their orthopaedic department which demonstrated similar deficient areas of operative note documentation, in particular the poor documentation of tourniquet time [4].

The important issue of legibility exists within all handwritten notes. It has been shown that using computer templates/proforma along with typed notes proves to be superior to handwritten notes [11]. However, when other staff members were asked to read notes, the problem of legibility arose. The use of electronic operation notes is currently being piloted by other surgical specialities in St. James's Hospital, with the aim that this will be available to orthopaedic surgery in the near future.

Electronic notes are beneficial in many ways. They can be accessed repeatedly and remotely from any hospital computer system. This eliminates the possibility of an operative note being lost or destroyed and markedly improves the notes in terms of detail and legibility [12]. The headings used in the notes not only can be standardised, but also can be edited to suit individual specialities, with specific headings and sections, as there is no need to print out standard proforma sheets. Electronic operation notes will become easier to audit and review for research purposes, as they are easier to access and will save the reviewer considerable time. Templates can also be added for common procedures so as to save time in the writing of an operation note and to guide trainees as to how a particular surgeon approaches a case or how they prefer their operation notes to be written and what information each note should contain.

A study conducted by the Centre for Disease Control and Prevention looked at the use of electronic patient records and showed that 74% of physicians highlighted the ability to access patient information as a benefit, along with 74% believing that electronic records had improved overall patient care [13].

Ghani et al. undertook a study piloting their “smart” electronic operation note system for orthopaedic trauma operation notes. They showed a marked improvement in the quality of documentation, both in terms of information detail and readability. The “smart” electronic notes were deemed to be completely legible (100%) compared with only 66% of the handwritten notes [12].

The 2014 Royal College of Surgeons Good Surgical Practice guidelines now state that all operation notes are “preferably typed.” This recommendation was not present in the 2008 guidelines and certainly favours a move towards electronic notes so as to be compliant with best surgical practice and patient care.

One issue remains is that some surgeons create illustrations in their operative notes to help explain certain complex issues. While this would be currently limited to electronic notes, the use of touch screen technology could provide a solution to this issue.

Limitations of this study included the small amount of operation notes collected between the allotted time periods. Ideally, a larger number of operation notes would have been collected. At the time of publication, the electronic operation notes are not available to the orthopaedic department, so a follow-up evaluation of the new electronic template was not possible. This would have allowed for a reaudit to assess any improvement in documentation with the electronic notes. While a standardised proforma exists in St. James's Hospital for all surgical procedures, an orthopaedic-specific proforma was not available. The operative notes audited were limited to those of inpatients in the orthopaedic ward during the specified time. This eliminated the day-case procedures and their operation notes, which would have increased the numbers of notes reviewed in the audit. Allowing for these limitations, the date collected shows areas of strength and weakness in St. James's Hospital orthopaedic operation note proforma and in the documentation of orthopaedic surgeries.

This data will allow for improvements to be made in documentation by the orthopaedic surgeons in the future. It highlighted the poor tourniquet time documentation, which can be improved upon, and also the need for prosthesis serial numbers to be documented, given the potential for future revisions or surgeries on the same patient.

## 6. Conclusions

The completion and documentation of surgical procedures on our standard St. James's Hospital operation sheets were excellent in terms of recording date, time, surgeon, closure details, procedure name, and signatures. Improvement is needed in documenting tourniquet time, prosthesis serial numbers, correct use of the template headings, incision, and operative diagnosis. These improvements could be made with the introduction of an orthopaedic-specific proforma with headings for tourniquet time, antibiotics, and prosthesis serial numbers.

Given the new RCSE guidelines recommendation for 2014, it is recommended that electronic notes be introduced in the orthopaedic department. As the electronic notes will be piloted in St. James's Hospital in the near future, it is our plan to audit those notes and compare them with the results we have obtained from the proforma sheets.
